# Clinical Challenges in Diagnosing Primordial Dwarfism: Insights from a MOPD II Case Study

**DOI:** 10.3390/medicina60111906

**Published:** 2024-11-20

**Authors:** Alexandru Daniel Jurca, Codruța Diana Petchesi, Sânziana Jurca, Emilia Severin, Aurora Alexandra Jurca, Claudia Maria Jurca

**Affiliations:** 1 Department of Preclinical Disciplines, Faculty of Medicine and Pharmacy, University of Oradea, 410081 Oradea, Romania; alexjurca@uoradea.ro (A.D.J.); petchesidiana@uoradea.ro (C.D.P.); claudiajurca@uoradea.ro (C.M.J.); 2 Regional Center of Medical Genetics Bihor, County Emergency Clinical Hospital Oradea (Part of ERN-ITHACA), 410469 Oradea, Romania; 3 Faculty of Medicine and Pharmacy, University of Oradea, 410081 Oradea, Romania; jurca.auroraalexandra@student.uoradea.ro; 4 Department of Genetics, ”Carol Davila”University of Medicine and Pharmacy–Bucharest, Dionisie Lupu Street, Number 37, District 2, 020021 Bucharest, Romania

**Keywords:** primordial dwarfism, MOPDII, PCNT gene, genetic variability, rare disease

## Abstract

*Background and Objectives.* **Primordial dwarfism (PD)** is a rare group of genetic conditions where individuals experience severe growth restriction, both in the womb and after birth. From as early as the fetal stage, those affected are significantly smaller than their peers. What makes PD distinct is its slow but steady growth pattern, resulting in proportionate dwarfism, where all parts of the body are equally shortened. Diagnosing and managing PD presents significant challenges due to its rarity and the wide range of clinical and genetic variability. The main conditions in this group include Seckel syndrome, Microcephalic Osteodysplastic Primordial Dwarfism (MOPD) types I/III, MOPD type II, Meier–Gorlin syndrome, and Silver–Russell syndrome (SRS). The first four—Seckel syndrome, MOPD types I/III, MOPD type II, and Meier–Gorlin syndrome—are associated with microcephaly, and together they are known as microcephalic PD. Given how uncommon PD is, establishing its exact incidence is difficult. It is estimated that about 4 million infants die within the first month of life, with 99% of these deaths occurring in the neonatal period. *Materials and Methods.* Accurately diagnosing PD requires meticulous evaluation, as it can be easily confused with other genetic disorders that also cause dwarfism. In this article, we present the case of a 10-year-old patient diagnosed with MOPD II, the most common and well-documented form of microcephalic PD. *Results*. Genetic analysis revealed a pathogenic variant in the **PCNT (pericentrin)** gene ((c.1550dup, p.Gln518Alafs*7), alongside a deletion of exons 37–41. *Conclusions*. This case sheds light on the clinical and genetic complexities of primordial dwarfism, underscoring the importance of timely and accurate diagnosis for effective patient care.

## 1. Introduction

Growth is a fundamental characteristic of all living organisms and represents a complex process that arises from a positive imbalance between cellular proliferation and cell death. Primordial dwarfism (PD) is a group of conditions marked by extremely reduced body size, which is evident from intrauterine life, persists at birth, and continues throughout life. Individuals affected by PD have significantly lower heights than the population average, typically presenting as harmonic dwarfism, where the body is well proportioned. This condition is associated with an increased risk of perinatal morbidity and mortality, affecting about 10% of pregnant women [1]. It is estimated that 4 million infants with PD die within the first four weeks of life, with 99% of these deaths occurring in the neonatal period [2,3].

Clinically, PD is divided into five main subtypes: Seckel syndrome (SS), Majewski/Microcephalic Osteodysplastic Primordial Dwarfism (MOPD) Types I/III, Type II, Meier–Gorlin syndrome (MGORS), and Russell-Silver syndrome (SRS). Most of these conditions are inherited in an autosomal recessive manner, although autosomal dominant inheritance or genomic imprinting can also occur [1,4]. The syndromes often exhibit overlapping clinical signs, making comprehensive clinical, imaging, and molecular evaluations vital for diagnosis.

While microcephaly is present in the first four subtypes—leading to the term “microcephalic primordial dwarfism”—the fifth subtype, SRS, presents with a normal head size according to WHO growth curves [5]. Diagnosing PD is challenging for clinicians due to several factors: the rarity and diversity of these conditions, their presence in intrauterine life, similarities to other disorders associated with short stature, and the complex genetic nature of the associated mutational variations. Furthermore, the multisystemic involvement in PD leads to various complications—including skeletal, cardiovascular, developmental delays, and dental issues—all appearing at different intervals, which further complicates diagnosis. Genetic counseling is essential for a better understanding of the disease.

This article aims to highlight the main clinical and genetic characteristics of PD, accompanied by a case illustration of MOPD II syndrome. A multidisciplinary team—including pediatricians, geneticists, endocrinologists, cardiologists, radiologists, psychologists, dentists, and others—plays a crucial role in monitoring and treating these patients. Additionally, the authors wish to explore the importance of publishing clinical and genetic data in the context of primordial dwarfism, emphasizing their impact on diagnosis and clinical management.

## 2. Materials and Methods

### 2.1. Case Report

The authors present the case of a 10-year-old patient, the first child in the family, who was diagnosed in utero with growth retardation. The patient was born at 37 weeks of gestation, with a birth weight of 1300 g and microcephaly. Growth retardation persisted after birth. Phenotypically, the patient exhibits an elongated and narrow face, a high-pitched voice, microtia (abnormal ear development), a prominent nose, and thin eyebrows. The family history is negative for chronic or genetic conditions.

### 2.2. Laboratory Investigations

Laboratory investigations (complete blood count, biochemical tests: liver and kidney function tests, hormonal tests: thyroid hormones, insulin, glucose, LH, FSH, growth hormone levels, and IgF1) were recommended to exclude other genetic syndromes with clinical signs overlapping those present in primordial dwarfism, such as Fanconi anemia, Bloom syndrome, and 3M syndrome [1,6].

### 2.3. Imagistic Investigations

Imaging studies used cranial X-Rays, skeletal imaging, ultrasound, and brain MRI.

### 2.4. Molecular Investigations

Written informed consent was obtained from the mother before participation in the study.

The first Next-Generation Sequencing analysis was performed in Timișoara in 2019 at the Regional Center for Medical Genetics (CRGM), using the MiSeq Illumina platform, which allows for the analysis of coding genomic DNA. The genomic DNA was fragmented, followed by the amplification of the coding sequences and library generation using the Illumina TruSight Cardio Sequencing panel (174 genes). Data analysis was conducted using the UCSC Genome Browser, OMIM, and DGV (Database for Genomic Variants), and the variants were interpreted according to ACMG guidelines.

Two years later, sequence analysis and deletion/duplication testing were performed, using the Invitae Skeletal Disorders panel (358 genes). DNA was extracted from the patient’s peripheral blood using standard extraction procedures. Genomic DNA obtained from the submitted sample was enriched for targeted regions using a hybridization-based protocol and sequenced using Illumina technology. All targeted regions were sequenced with ≥50x depth. Exonic deletions and duplications were called using an in-house Invitae algorithm that determines the copy number at each target by comparing the read depth for each target in the proband sequence with both the mean read depth and the read depth distribution obtained from a set of clinical samples. Data analysis was conducted using PMID (Pub Med), rsID (Reference SNP ID), MedGenID identifier, and OMIM, and the variants were interpreted according to ACMG guidelines. The confirmation of the presence and location of reportable variants was performed based on stringent criteria established by Invitae (1400 16th Street, San Francisco, CA 94103, #05D2040778), as needed, using one of several validated orthogonal approaches (PubMed ID 30610921).

Sequence analysis and deletion/duplication testing of the two variants were then performed in both parents using Invitae Laboratory sequence analysis and deletion/duplication testing.

## 3. Results

### 3.1. Clinical Evaluation of the Patient Land Laboratory Investigations

**Figure 1:**
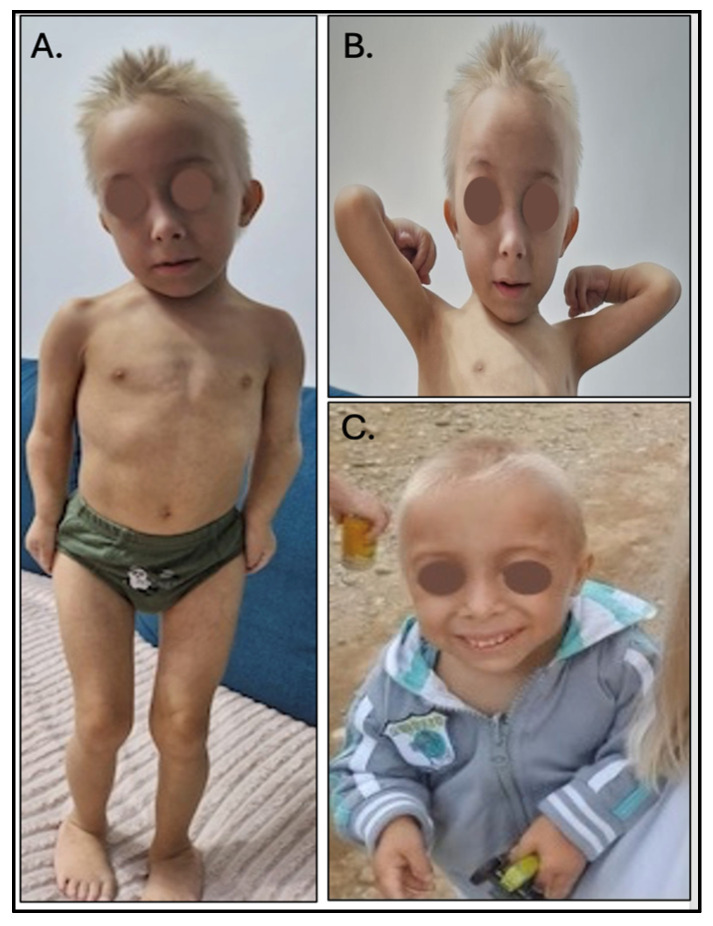
The clinical findings in a 10-year-old male patient (**A**): Anterior view of the 10-year-old patient showing all body segments shortened symmetrically; (**B**,**C**): Frontal view of the patient’s face revealed an elongated face, mild prominence of the eye globes (slight maxillary protrusion), a small chin (micrognathia), and dental anomalies involving both position and size.

**Figure 2:**
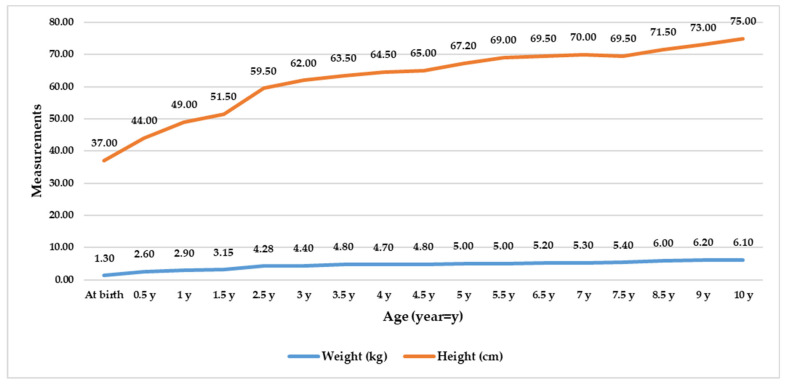
The patient exhibits significantly reduced growth velocity compared to his peers.

The patient is 10 years old, with an extremely small stature and weight. Height: 75 cm (Z-index −10.62, RO); weight: 6.1 kg (Z-index −28.4, RO). Craniofacial dysmorphism: microcephaly, light blond phenotype, elongated face, slight maxillary protrusion of the eye globes, smaller chin, dental positional, and size anomalies (Figure 1). The patient presented with a high-pitched voice. The dwarfism is proportionate, with all body segments shortened symmetrically. The extremely slow growth velocity can be observed in Figure 2.

### 3.2. Laboratory Investigations

Biochemical, hematological, and hormonal (thyroid hormones) investigations revealed normal values.

### 3.3. Imaging

#### X-Rays

Table 1 describes the evolutionary radiological changes over the years and two recent skeletal radiographs (head, chest, and limbs). The wrist X-Rays revealed delayed bone maturation and skeletal abnormalities such as radial head dislocation or shortened metacarpal bones.

### 3.4. Molecular Investigations

The first molecular test (TrueSight One gene panel, CRGM Timiș, 2019) revealed a pathogenic variant in the *PCNT* gene, c1550dup (p.GLN518Alafs*7). In 2021 (Invitae laboratory), through panel sequence analysis and a deletion/duplication in-house protocol in addition to the previously described pathogenic variant, a likely pathogenic deletion of exons 37–41 in the *PCNT* gene was found.

Sequence analysis and deletion/duplication testing of the two variants performed in both parents revealed the maternal origin for the pathogenic variant of the *PCNT* gene, c1550dup (p.GLN518Alafs*7), and the paternal origin for the deletion of exons 37–41 of the *PCNT* gene.

## 4. Discussions

### 4.1. Clinical Overview and Genetics of Primordial Dwarfism

#### 4.1.1. Seckel Syndrome

Seckel syndrome is an extremely rare syndrome with autosomal recessive inheritance, affecting 1 in 10,000 children [7]. Approximately 100 cases have been reported in the literature [8]. Its main features include severe prenatal growth retardation that persists postnatally. The growth rate is extremely low, with the final height of patients being around 145–157 cm, while the arms and legs are proportionate to their height. Craniofacial dysmorphism is present and includes microcephaly, a narrow forehead with posterior inclination, a narrow face, strabismus, antimongoloid orientation of the palpebral fissures, low-set ears, a wide nose with a beak-like appearance, an ogival palate, and dental anomalies in number, size, and micrognathism. Skeletal changes may occur, including delayed bone age, pectus carinatum/excavatum, kyphoscoliosis, radial dislocation, or dislocation at the elbow and knee joints, hip dysplasia, and congenital clubfoot. Changes in the external genitalia may be observed: in males, cryptorchidism occurs, while in females, clitoromegaly may be present in some cases. Moderate intellectual disability can be encountered in some patients [9,10,11,12].

Seckel syndrome (SS) is a genetically heterogeneous condition. A variety of genes are involved in its etiology, most of which play a role in the DNA repair response. Although first described in 1960 by Seckel, the first locus associated with the occurrence of SS, *SCKL1*, was only identified in 2000 on chromosome 3 (3q22.1-q24), where the gene encoding the ataxia–telangiectasia and Rad3-related protein (*ATR* gene) is located. This gene plays an essential role in the cellular response to DNA damage and is crucial in maintaining genomic stability. The protein produced is a kinase that detects DNA breaks and activates repair processes, particularly during DNA replication. As such, mutations in this gene result in defects in DNA repair mechanisms, significantly affecting cellular growth [13,14].

In 2001, a second locus, *SCKL2*, was identified on chromosome 18 (18p11.31-q11.2), and, in 2023, Mudassil et al. identified the *RTTN* gene on the same chromosome 18 (18q22.2), whose mutations can also cause Seckel syndrome [15]. The *RTTN* gene encodes a protein called rotatin, which is involved in cell division and tissue development, including the brain. Mutations in this gene result in defects in microtubule organization, affecting mitosis and causing issues in embryonic development. The third locus, *SCKL3*, was identified in 2003 on chromosome 14 (14q23) [16,17].

To date, at least 10 genes have been identified in its pathogenesis: *ATR* [18], *RBBP8* [19], *CEP152* [20], *CENP (CPAP)* [21], *CEP63* [22], *DNA2* [23], *ATRIP* [24], *Ninein (NIN)* [25], *PLK4* [26], and *CDK5RAP2* [27]. Mutational variations in these genes disrupt the balance between cellular proliferation and apoptosis, leading to a reduction in total body mass, as observed in primordial dwarfism with microcephaly (MPD) [28].

#### 4.1.2. MODP Types I, III

These are extremely rare diseases. They are variations of a single type, and thus they are classified together. Phenotypically, they are characterized by severe growth retardation, brain anomalies, dry skin, microcephaly, thin and fragile hair, thin eyebrows, short and thin vertebrae, femoral dislocation, and clavicular anomalies. Seizures, apnea episodes, and agenesis of the corpus callosum may occur [29].

In MOPD I/III (Taybi–Linder syndrome), the gene that plays a primordial role is RNU4ATAC, which has a very simple structure consisting of only 130 nucleotides [30]. Mutational variants in this gene are responsible for the occurrence of MOPD I/III, Roifman syndrome (RS), and Lowry–Wood syndrome (LWS), all of which exhibit diverse phenotypic heterogeneity with certain overlapping clinical signs [31,32]. The RNU4ATAC gene does not code for proteins but for small nuclear RNA (snRNA), which is part of the U12-dependent minor spliceosome complex involved in the splicing of a subset of introns. Thus, the normal splicing process is disrupted, leading to extensive effects on gene expression, which contributes to the severe developmental anomalies observed in individuals with this syndrome.

#### 4.1.3. MOPD II Syndrome

MOPD II syndrome is distinct from types I and III and is considered a completely different syndrome with autosomal recessive inheritance. It is estimated to be the most common form of primordial dwarfism [33]. The clinical picture is characterized by intrauterine growth retardation, extremely low birth weight, and a high-pitched voice; craniofacial dysmorphism includes microcephaly, a narrow face with a prominent nose and bulging eyes and dental anomalies in size (microdontia) and number (oligodontia); skeletal features include delayed bone age, fragile and thin bones, a small winged ilium, coxa vara deformity, and hip dislocation; scoliosis may also be present; cognitive function can range from borderline to moderate intellectual disability; total body size is greatly reduced, with a final height around 110 cm [33]. Notably, cerebrovascular complications may arise: moyamoya disease or cerebral aneurysms, and changes in the complete blood count, such as the presence of anemia, leukocytosis, or thrombocytosis, in some patients [34]. Therefore, monitoring these patients necessitates screening for cerebrovascular anomalies: brain MRI and angiography MRI at intervals of 12–18 months. Annual MRI is recommended until the age of 10 [34]. The average life expectancy is approximately 30 years (the oldest confirmed molecular patient was 39 years old); thus, early diagnosis and the proper monitoring of these patients are crucial to improving their quality of life and increasing their life expectancy [35,36]. Our patient diagnosed with this syndrome presents phenotypically with characteristics similar to other patients described in the literature with this syndrome; the complete blood count revealed the presence of hypochromic anemia, with a hemoglobin value of 10 g/dL. X-rays of the upper and lower limbs indicated changes in structure and bone density (see Table 1). Repeated brain MRIs did not reveal pathological changes, and moyamoya disease was not detected.

MOPD II syndrome is distinguished from types I and III and is considered a completely different syndrome with autosomal recessive inheritance. It is estimated to be the most common form of primordial dwarfism [33]. This syndrome arises from mutational variants present in the PCNT gene located on the long arm of chromosome 21 (21q22.3). This gene codes for a protein called pericentrin, which plays a major role in anchoring structural and regulatory components at the centrosome. The mutations lead to chromosomal disintegration by disorganizing the mitotic spindle [37,38]. As such, cell division (mitosis) is disrupted, resulting in reduced cellular production and accelerated apoptosis, leading to the characteristic clinical presentation of MOPD II syndrome. Our patient diagnosed with this syndrome phenotypically presents characteristics similar to other patients described in the literature with this syndrome. This association of a frameshift mutation, c.1550dup (p.Gln518Alafs*7), with the appearance of a premature stop codon and a deletion of exons 37–41 in the PCNT gene has not been reported in the literature to our knowledge.

#### 4.1.4. Meier–Gorlin Syndrome—MGS (Ear–Patella–Short Stature Syndrome)

This is the fourth subtype of primordial dwarfism characterized by short stature, microcephaly, bilateral microtia, and patellar hypoplasia/aplasia, but without intellectual disability; it may also be associated with hearing loss, cryptorchidism, and bone malformations (ribs, clavicles) [39,40,41].

MGS is also characterized by genetic heterogeneity. A total of five genes are involved, which participate in the DNA replication mechanism. The currently identified genes are ORC1 [Origin Recognition Complex 1], ORC4, ORC6, CDT1, CDC6, and CDC45. Mutational variants described in any of these genes are found in 65–78% of patients [1,36,42].

#### 4.1.5. Russel–Silver Syndrome

This represents the fifth subtype of primordial dwarfism and is the only syndrome in which microcephaly is absent. The main features include low birth weight, short stature, or the presence of body asymmetry, though not in all patients. In terms of craniofacial dysmorphism, they exhibit a triangular face, a prominent forehead, dental anomalies, and a small mandible [43,44]. Gastrointestinal problems such as feeding difficulties, gastroesophageal reflux, and vomiting, which together promote malnutrition, are common signs and may sometimes be the first indicators that alert parents [45].

Table 2 provides a synoptic summary of the clinical features of the five types of primordial dwarfism (PD).

The genetic changes encountered are diverse. Multiple chromosomal alterations can occur, leading to increased diversity (like a wide range) [1,46,47]. As early as 2010, Eggermann et al. demonstrated changes on chromosome 11p15, specifically the presence of duplication at this level; in 2012, Coutton et al. highlighted the duplication present on the short arm of chromosome 17, a region known for its gene content implicated in the pathogenesis of SRS [48,49]. Other authors, such as Lin et al. and Spengler et al., reported the presence of microdeletions on chromosomes 1, 7, 13, 14, 12, and 15. The literature has also reported cases of reciprocal translocations, such as between chromosomes 17 and 1: t(17;20)(q25;ql3) [43,50,51,52] or between 11 and 16: t(11;16)(p13;q24.3) as reported by Rao et al. [43]. Fifty percent of cases of SRS are caused by the maternal uniparental disomy of chromosome 7 or genomic imprinting defects, specifically the hypomethylation of the H19 gene. Nonetheless, a high percentage of cases (some authors suggest that 50%) remain without a precise etiology. Cases of the dysregulation of 11p15 have also been reported, along with the occurrence of uniparental disomy that does not involve chromosome 7 or methylation changes outside the imprinted gene loci region of 11p15, or other methylation anomalies occurring at other loci [53]. New cis-acting and trans-acting factors have also been reported, which regulate the imprinting center of 11p15, the hypomethylation of the telomeric imprinting center ICR1, and small copy number variations [54].

### 4.2. Differential Diagnosis

The clinical picture of primordial dwarfism overlaps with that of other genetic syndromes [55], making accurate clinical and radiological evaluation extremely important. Molecular diagnosis is essential for establishing a definitive diagnosis (Table 3).

### 4.3. Treatment of PD

The main issue in primordial dwarfism is short stature; thus, the major objective of treatment is to improve the growth curve; unfortunately, no specific treatment exists for this [1,56,57].

Management focuses on monitoring and addressing other signs and symptoms that may arise, such as nutritional support, hormone therapy, and surgical interventions for specific complications. Growth hormone treatment has minor effects and is clearly recommended only for Silver–Russell syndrome. Some authors, such as Birkebaek, have shown the positive effects of GH in cases of Seckel dwarfism, while Munnich et al. have noted benefits in Meier–Gorlin syndrome [58,59]. Conversely, growth hormone therapy has no effect on individuals diagnosed with MOPD I/III or MOPD type II. Studies have demonstrated no significant difference between MOPD II patients treated with GH and those who did not receive this treatment. However, it seems that this treatment targets the associated complications and comorbidities. More studies are needed to prove the efficacy of growth hormone treatment in patients with primordial dwarfism [6]. If moyamoya complications are detected, revascularization procedures such as encephalodurosynangiosis (EDAS) are performed. If insulin resistance occurs, oral antidiabetic medications are administered, and if secondary hypertension due to renal complications arises, antihypertensive drugs are given. The patient presented by the authors with MOPD type II received GH treatment for 12 months before molecular diagnosis confirmation, but no improvement in the growth curve was observed, leading to its cessation. Periodic dental check-ups were conducted to assess and possibly intervene surgically.

### 4.4. Diagnosis and Monitoring

Clinical differences in patients with primordial dwarfism (PD) may be attributed to mutations in various genes. Diagnosis may require long-term observation to track growth patterns and the development of symptoms over time, which may delay the identification of the specific disorder. Genetic testing can confirm the specific type of primordial dwarfism. Diagnosis may also involve assessing growth patterns, physical examination, and imaging studies to evaluate skeletal development. Generally, in all subtypes of PD, but especially in microcephalic primordial dwarfism, monitoring involves periodic reassessments to track the emergence of skeletal changes: coxa vara deformity (observed at a young age), scoliosis, and pelvic dislocation. Cardiological evaluation and imaging to highlight vascular complications are also important. Additionally, periodic laboratory investigations are recommended, such as complete blood count, careful monitoring of blood glucose to timely identify possible insulin resistance, and hepatic and renal function assessments; periodic evaluation of growth curves is necessary, as affected individuals have a much slower growth rate compared to the general population. Wilems et al., in a study that included 25 patients, with MOPD II emphasized that they form a homogeneous group, sharing similar characteristics in terms of clinical, genetic, and disease progression traits [60]. This homogeneity can facilitate a deeper understanding of the condition and contribute to the development of more tailored and effective treatment strategies for these patients. In 2023 Duker et al., in a review paper, showed that there are no genotype–phenotype correlations [61]. The case presented by the authors resembles the others described in the literature, following the natural progression of the disease.

### 4.5. Genetic Counseling

MOPDII is an autosomal recessive disorder. In our patient’s case, both parents are carriers for a mutational variant, one pathogenic and the other one likely pathogenic; they have a 25% chance of having another affected child, 25% chance of having a carrier child for the pathogenic variant, 25% chance of having a carrier child for the likely pathogenic variant, and 25% chance of having a healthy, non-carrier child. The advantage of identifying the underlying mutations is the possibility of a prenatal diagnosis for each of the following pregnancies [37,60].

## 5. Conclusions

Primordial dwarfism is a very rare and complex condition that poses a significant diagnostic challenge for clinicians. This complexity is important for effectively managing the health and developmental needs of affected persons. Early diagnosis, coupled with advancements in screening techniques and prenatal diagnostic methods, can significantly enhance the management of these rare disorders. Identifying primordial dwarfism early not only improves patient outcomes but also provides essential support to families through informed genetic counseling. These efforts, when integrated, can improve the quality of life and provide hope for those affected by this rare condition.

## Figures and Tables

**Figure 3:**
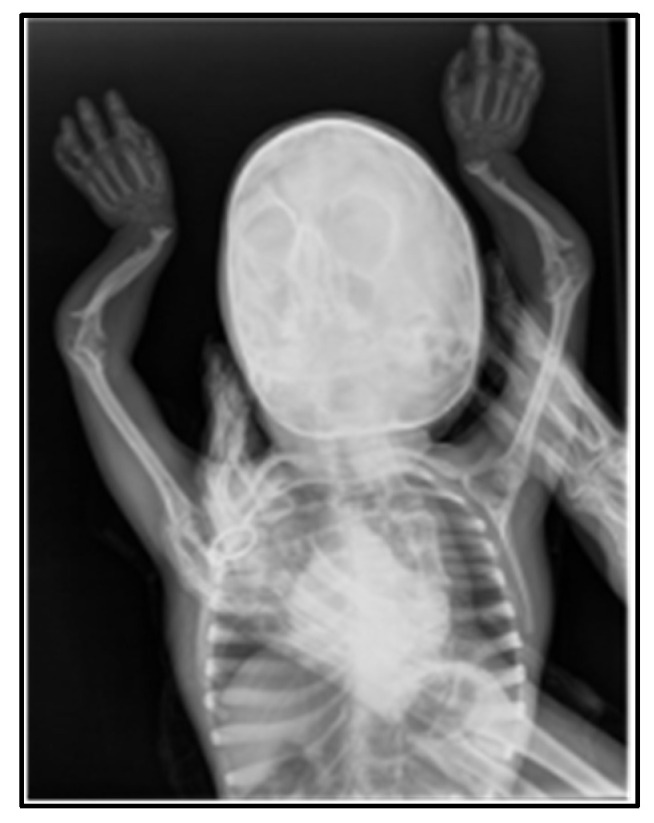
Radiological changes observed: widened, flared ‘champagne glass’ metaphyses with irregular contours in the forearm bones bilaterally. The radial and ulnar diaphyses are curved bilaterally. The left radius is shortened, with flared and irregular metaphyses in the distal phalanges and metacarpals.

**Figure 4:**
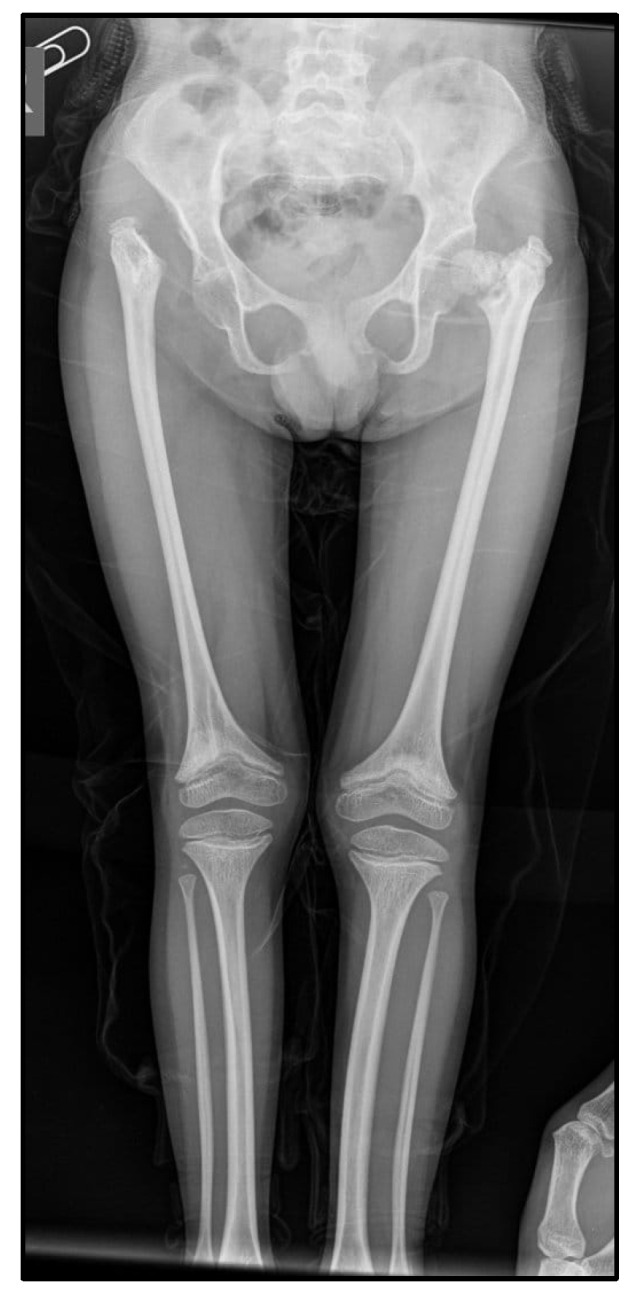
Radiological changes observed in the pelvis and femora: the right acetabular cavity is flattened and vertically oriented. The femoral head nucleus on the right is not visible, and the right femur is elevated. The left acetabular cavity is slightly vertically oriented. The left femoral head nucleus is formed but irregular and fragmented. Bilateral femoral metaphyses are widened, flared, and irregular. Bilateral tibial metaphyses are also widened and flared.

**Table 1:** Evolutionary radiological features from birth to ten years in a patient with primordial dwarfism (PD).

Year		Skull	Upper Limbs	Lower Limbs	Wrist Bone Age
	Bone Region:				
2014 (year of patient’s birth)		Slightly blurred coronal sutures in the middle third. Sella turcica is normal in size. Dysmorphic appearance of the orbits.	Absence of carpal ossification nuclei, corresponding to newborn bone age. Altered appearance of the distal radioulnar metaphyses, with widening and deepening of their concavities—rickets suspected?		
2015		Oval transparent zones (possible lack of mineralization or osteolysis?) in the parieto-occipital and frontal areas.	Suggestive of rickets: absence of carpal growth nuclei, osteotransparency, curved diaphyses with prominent, widened metaphyses (“champagne glass” shape), no visible bone cortex in the forearm bones.	Cortex not visible in the lower limbs, particularly in the femoral region.	
2017		No significant radiological changes in the cranial bones. Sella turcica has a normal radiological appearance.	The radius is slightly curved, with mild osteosclerosis in the distal metaphysis. The distal ulnar metaphysis shows a slightly concave appearance.	Osteopenia, asymmetric pelvis, vertically oriented, unformed acetabular cavity, more pronounced on the right side. Femoral nuclei are undersized for the patient’s age. Bilateral hip shows changes suggestive of hip dysplasia, with no significant structural bone changes. Delayed bone development. In the bilateral feet, the middle phalanx nuclei are small. Osteopenic appearance of visualized bone segments.	Bone age = 8 months
2021					Bone age = 2 years
2024		Partial empty sella (October 2024)	Widened, flared “champagne glass” metaphyses with irregular contours in the forearm bones bilaterally. The radial and ulnar diaphyses are curved bilaterally. The left radius is shortened (4.5 cm versus 5.8 cm on the right). Ossification nuclei are present in the hand skeleton: trapezium, trapezoid, capitate, hamate, and lunate. The scaphoid nucleus is very small (~2 mm); the distal radial epiphysis nucleus is rudimentary—bone age of 4–5 years. Flared and irregular metaphyses in the distal phalanges and metacarpals (Figure 3).	The right acetabular cavity is flattened and vertically oriented. The femoral head nucleus on the right is not visible, and the right femur is elevated. The left acetabular cavity is slightly vertically oriented. The left femoral head nucleus is formed but irregular and fragmented. Coxa vara. Bilateral femoral metaphyses are widened, flared, and irregular, with an “inverted V” appearance of the growth line. Bilateral tibial metaphyses are also widened and flared (Figure 4).	Bone age = 4–5 years

**Table 2:** Clinical aspects of the 5 types of primordial dwarfism.

Subtypes	Circumference of the Head	Phenotype
Seckel syndrome	Microcephaly	Narrow face, dental anomalies (number, position, size), nose with a prominent tip resembling a “beak”, retrognathism.
Majewski/Microcephalic osteodysplastic primordial dwarfism (MOPD) types I/III		Rare hair and eyebrows, dry skin.
MOPD type II		Prominent nose, slightly protruding eyes (exophthalmos), small chin Dental anomalies (size: small teeth; number: oligodontia), high-pitched, shrill voice.
Meier–Gorlin syndrome (ear–patella–short stature syndrome)		Microtia Underdeveloped ears, absent/hypoplastic patellae.
Silver–Russell syndrome	Normal-sized skull	Small triangular face, micrognathia, dental anomalies.

**Table 3:** Genetic syndromes with clinical signs overlapping primordial dwarfism.

Syndrome	Common Features	Differentiated Features	Overlapping Subtype Features
**3M syndrome** **autosomal recessive (AR)** ICD-11: LD24.D ORPHA:2616 OMIM: 273750 612921 614205	Prenatal and postnatal growth retardation, facial dysmorphism: prominent chin giving a triangular appearance, clinodactyly, joint laxity, thin tubular bones.	Hemihypertrophy absent, more severe dwarfism both clinically and radiologically; tall and thin vertebrae; much smaller pubic bones.	Silver–Russell syndrome
**Fanconi anemia** **autosomal recessive (AR)** ICD-11: 3A70.0 ORPHA:84 OMIM: 617883 617243 617244 617247 227645 227646 227650 300514 600901 603467609053 609054 610832 613390 613951614082 614083 615272 616435	Short stature with growth retardation; facial dysmorphism, skin hyperpigmentation with café au lait spots; bone anomalies: absent radius, polydactyly malformation; aplastic anemia with evolving bone marrow failure; predisposition to leukemia; mutations in ATR genes.	Hematologic profile is much more severe; an increased number of genes are involved: currently 21.	Seckel syndrome
**Donohue syndrome** (LEPRECHAUNISM) **autosomal recessive (AR)** ICD-11: 5A44 ORPHA:508 OMIM: 246200	Prenatal and postnatal growth retardation, low-set ears, absence of subcutaneous tissue, muscle hypotonia, hypoglycemia, acanthosis nigricans.	Insulin receptor defect.	Silver–Russell syndrome
**Nijmegen breakage syndrome (NBS)** (Ataxia-telangiectasia, variant 1; Berlin breakage syndrome; Immunodeficiency-microcephaly-chromosomal instability syndrome; Microcephaly-immunodeficiency-lymphoid malignancy syndrome; Seemanova syndrome type 2) **autosomal recessive (AR)** ICD-11: 4A01.31 ORPHA:647 OMIM: 251260	Prenatal and postnatal growth retardation, craniofacial dysmorphism (microcephaly, inclined forehead, prominent midface, large ears, café au lait spots).	Mutation in the NBN gene ((8q21-q24) specifically within exons 6–10, which leads to partially functional truncated fragments of nibrin, the gene product involved in repairing DNA double-strand breaks.	Seckel syndrome
**Cornelia de Lange syndrome** **(Brachmann-de Lange syndrome)** **autosomal dominant**, not applicable, X-linked recessive ICD-11: LD2F.1Y ORPHA:199 OMIM: 122470 300590 300882 610759 614701	Growth retardation; craniofacial dysmorphism: microcephaly, grotesque facial appearance, arched eyebrows fused at the midline (synophrys), elongated philtrum, thin lips; intellectual disability.	Seven genes involved in etiology, but *NIPBL* variants (5p13.2) are the most common cause (70% of patients).	Seckel syndrome
**Bloom syndrome (Bsyn)** **autosomal recessive (AR)** ICD-11: 4A01.31 ORPHA:125 OMIM: 210900	Prenatal and postnatal growth retardation; craniofacial dysmorphism: microcephaly, pigmentation disorders, high-pitched voice.	Malar hypoplasia, facial telangiectasias, photosensitivity pigmentary anomalies, increased risk of malignancies, immune deficiency, chromosomal breaks.	MOPD

## Data Availability

Data are contained within the article.
